# Opioid-Free Anesthesia Versus Opioid-Balanced Anesthesia in Breast Surgeries: A Randomized Study

**DOI:** 10.7759/cureus.84034

**Published:** 2025-05-13

**Authors:** A Chaitanya Pratyusha, Rama Krishna Prasad CH, Sandeep Garre, Kalyani Sangineni, Syama Sundar Ayya, Sushmita Salian, Bhargav Ram

**Affiliations:** 1 Anaesthesiology, All India Institute of Medical Sciences, Bibinagar, Hyderabad, IND

**Keywords:** analgesics, fentanyl, opioids, postoperative nausea and vomiting, visual analogue scale

## Abstract

Introduction

Opioids, the conventional analgesics, have adverse effects such as postoperative nausea and vomiting (PONV). The opioid-free anesthesia (OFA) protocols are now being formulated to provide equally efficacious analgesia with reduced adverse effects as opioid-balanced anesthesia (OBA). The primary objective was to compare the Quality of Recovery score (QoR-15 score) and intraoperative hemodynamic parameters between OFA and OBA. The secondary objectives were to compare the visual analog scale (VAS) scores, the number of patients requiring rescue analgesia, and adverse effects.

Methodology

Forty-eight patients undergoing breast surgery were randomly allocated to either the OFA or OBA group. A thoracic paravertebral block with 0.2% ropivacaine, followed by general anesthesia without opioids, was used in the OFA group. A transdermal fentanyl patch was applied 10 hours before induction of general anesthesia in the OBA group. QoR-15 scores at 24 and 48 hours postoperatively, intraoperative hemodynamic parameters, VAS score for 48 hours, and adverse events were noted. Data was represented as the median and interquartile range (IQR). The Mann-Whitney test was used for continuous variables and Fisher’s exact test was used for categorical variables.

Results

The median (IQR) QoR-15 score was 130 (128-132.75) in the OBA group and 132.5 (132-135) in the OFA group (p = 0.054) at 24 hours and 142 (141-145) in the OBA group vs 145 in the OFA group (140.25-146) (p = 0.367) at 48 hours. QoR-15 in the OFA group had a higher median (IQR) for physical independence, 17.5 (16-18), against 16 (16-17) in the OBA group, with a p-value of 0.016. Four patients in the OBA group had PONV and none in the OFA group (p = 0.037). The comparison of VAS scores and hemodynamic parameters at all the time points was insignificant.

Conclusion

OFA is similar to OBA, considering the overall quality of recovery according to the QoR-15 score, postoperative analgesia, and intraoperative hemodynamic stability with decreased incidence of PONV.

## Introduction

Opioids are conventional drugs used for intraoperative pain relief in general anesthesia. Despite their effectiveness, they are associated with a risk of nausea, respiratory depression, ileus, hyperalgesia, and the development of tolerance. Breast surgery under general anesthesia is associated with a 30%‑40% incidence of postoperative nausea and vomiting (PONV) and acute postoperative or chronic debilitating pain [1].

A systematic review of 23 randomized trials suggests that opioid-free anesthesia (OFA) may decrease the incidence of PONV without altering pain control when compared to opioid-balanced anesthesia (OBA) [2]. Various methods were described in the literature on OFA in breast surgeries which include the use of alternate intravenous drugs such as lignocaine, ketamine, and dexmedetomidine, or the addition of regional anesthesia techniques like paravertebral block and erector spinae block which can reduce the neuroendocrine stress response during surgery, reduce the opioid requirements thereby reducing opioid-related side effects such as nausea and vomiting, and a shorter duration of hospital stay [3,4]. As an alpha agonist, dexmedetomidine exhibits anxiolytic, sympatholytic, and analgesic properties, helping to minimize postoperative pain, reduce opioid use, and lower the incidence of opioid-related adverse effects [5].

Our primary objective was a comparison of the Quality of Recovery score (QoR-15 score), a validated patient-reported questionnaire [6] and intraoperative hemodynamic parameters between opioid-free general anesthesia with thoracic paravertebral block and opioid-balanced general anesthesia with a transdermal fentanyl patch. The secondary objectives included comparing the pain scores by the visual analog scale (VAS), the number of patients requiring rescue analgesia, and adverse effects among the two groups.

## Materials and methods

Methodology

This prospective randomized study was conducted in a tertiary care institute between December 2022 and March 2024, following the good clinical practice guidelines and abiding by the Declaration of Helsinki. The trial was registered under CTRI with registration number CTRI/2022/12/048052 after obtaining the institutional ethical committee approval numbered AIIMS/BBN//IEC/OCT/2022/222 dated 17-10-2022. Informed consent was taken from all the eligible participants for inclusion in the study.

A computer-generated randomization sequence was prepared by Dr. SG, using RAND (01) (Microsoft 2010) (Microsoft Excel, Microsoft Corporation, Redmond, Washington), and a sequentially numbered, sealed envelope technique was employed to maintain allocation concealment.

Dr. SS performed group allocation of the study participants based on the envelopes, to either of the two groups, namely, Group OFA to receive general anesthesia with thoracic paravertebral block and Group OBA to receive general anesthesia with a fentanyl patch and was responsible for intraoperative anesthetic management. Dr. RK, who was responsible for outcome assessment, was blinded to group allocation and was not involved in the anesthetic management. Trends in intraoperative hemodynamic parameters were noted from the monitor at the end of the surgery.

Consenting patients between the ages of 18 to 70, belonging to an American Society of Anesthesiologists (ASA) physical status I to III undergoing modified radical mastectomy were included. Exclusion criteria included emergency surgeries, bleeding disorders, anti-coagulants, chronic opioid use, allergy to the study drugs, infections, and lesions at the puncture site for the paravertebral block. The QoR-15 and VAS scores were explained to all the patients during the pre-anesthetic evaluation.

A transdermal patch of fentanyl 25 micrograms was applied in the left infraclavicular region in Group OBA 10 hours before the planned surgery, and general anesthesia was induced with fentanyl 2 microgram/kg, propofol 2-3 mg/kg, and atracurium 0.5mg/kg. 0.2mg/kg of fentanyl was administered for a heart rate or mean arterial pressure change of more than 20% from baseline during the intraoperative period.

Group OFA received the paravertebral block before induction of general anesthesia, with a 19-G 10cm nerve block needle using a 6-13 MHz linear USG probe (Edge II, Fujifilm Sonosite. Inc, Worldwide Headquarters, Bothell, WA, USA) at the T4-T5 vertebral level with a bolus of 20 ml of 0.2% ropivacaine. A 20-G catheter was left in the paravertebral space for postoperative use. A loading dose of 1 microgram/kg dexmedetomidine was given as an infusion over 10 min, followed by induction with propofol 2-3 mg/kg and atracurium 0.5mg/kg. A maintenance dose of 0.3 microgram/kg/hour of dexmedetomidine was continued throughout the procedure. 0.25 mg/kg Ketamine was given just before the skin incision. Ketamine 0.15mg/kg was given for a heart rate or mean arterial pressure change of more than 20% from baseline during the intraoperative period. 10 ml 0.2% ropivacaine was administered through the catheter in the paravertebral space 4th hourly for 72 hours after surgery.

In both groups, anesthesia was maintained with isoflurane at a minimum alveolar concentration of 0.8 to 1.2. After induction, 15mg/kg intravenous paracetamol was administered to all the patients, 4mg intravenous ondansetron was given 30 minutes before extubation, and the neuromuscular block was reversed with 50 μg/kg neostigmine and glycopyrrolate 10 μg/kg. Intraoperative heart rate and mean arterial pressures at baseline, immediately after incision, at 15-minute intervals till one hour, and 120 minutes were collected by Dr RK from the data saved in the monitor. The VAS was assessed at the end of surgery, one hour, two hours, six hours, 12 hours, 24 hours, and 48 hours postoperatively. The QoR-15 score was assessed 24 hours and 48 hours after surgery. All the patients received paracetamol 1gm BD for 24 hours postoperatively. 50mg intravenous tramadol was primary rescue analgesia if VAS was more than three, and 3mg intravenous morphine was given as secondary rescue analgesia if VAS continued to be more than three after 30 minutes. The total analgesic consumption in 24 hours, time for 1st rescue analgesia, and adverse events were noted.

Based on a mean difference of a QoR-15 score of 10 and a standard deviation of 11.2 obtained from a previous study [7], to achieve 80% power at a 5% significance level with a margin of equivalence of 10% (±0.1 units), the study would require a sample size of 22 per group, totaling 44 participants with equal group sizes. The total sample size was estimated to be 48, considering a 10 % loss of followers or patient dropout.

IBM SPSS Statistics for Windows, Version 23 (Released 2015; IBM Corp., Armonk, New York, United States) was used for statistical analysis. Box plots with whiskers were generated using Microsoft Excel. Non-parametric distribution was assumed since the sample size was less than 30 in each group and the data was represented as median and interquartile range (IQR). The Mann-Whitney test was performed for continuous variables, and for categorical variables, the chi-square test was utilized. A p-value less than 0.05 was considered significant.

## Results

**Figure 1:**
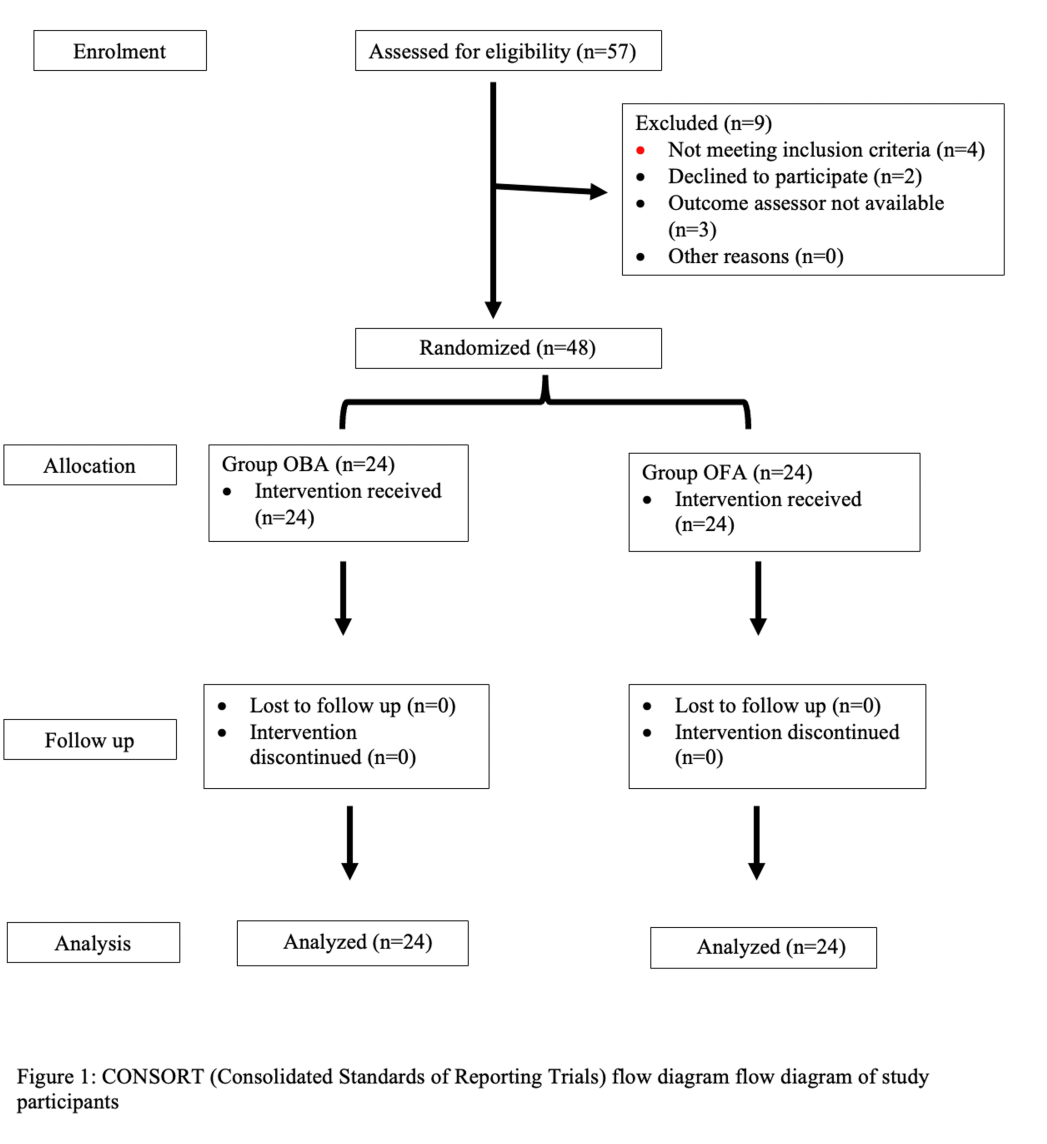
CONSORT (Consolidated Standards of Reporting Trials) Flow Diagram of Study Participants OBA: Opioid-Balanced Anesthesia; OFA: Opioid-Free Anesthesia

**Figure 2:**
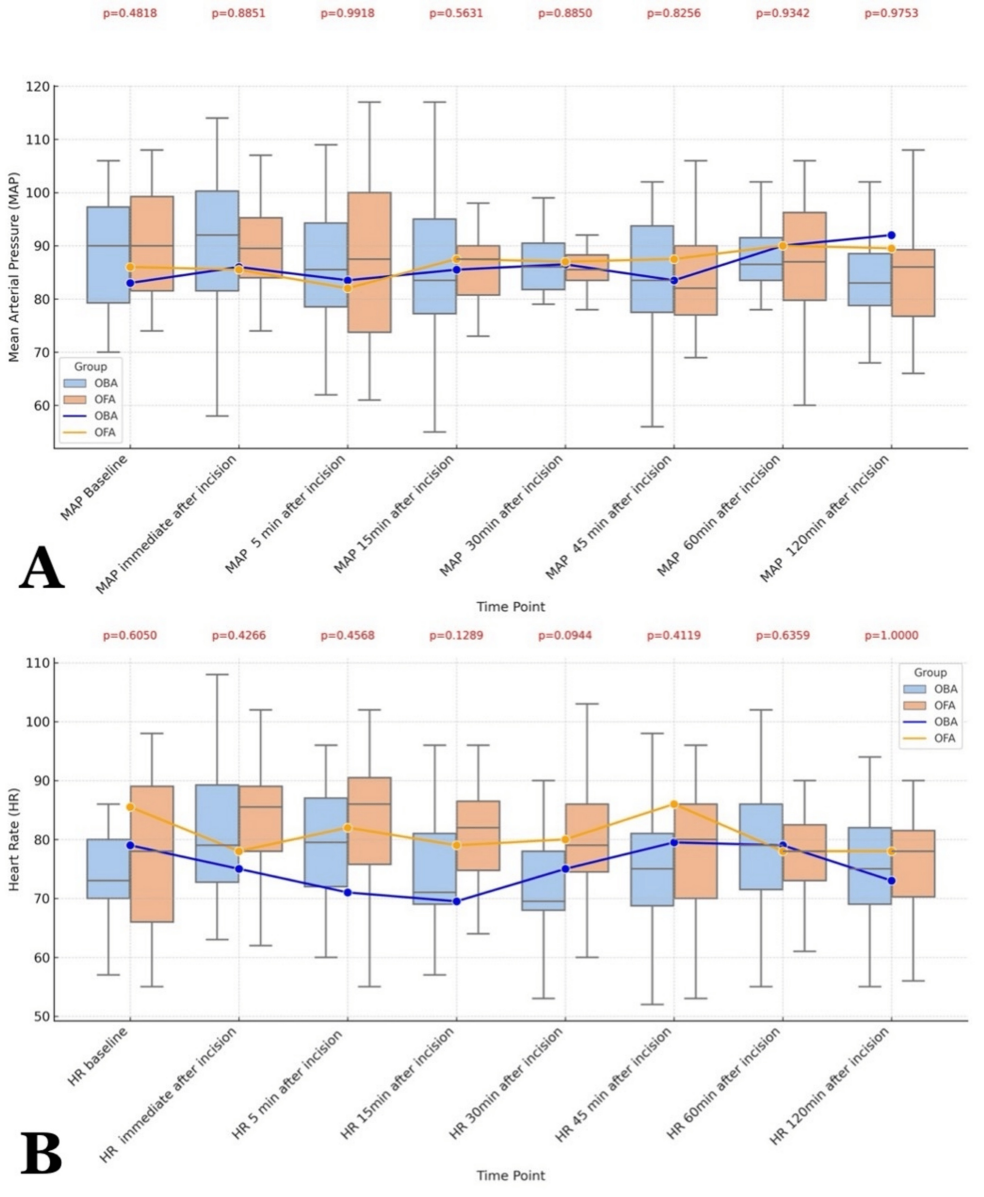
Comparative Analysis of Hemodynamic Changes Between OBA and OFA from the Baseline to Two Hours After Incision A: Mean Arterial pressure at different time points B: Heart rate at different time points A Mann-Whitney U test was applied, and a p-value less than 0.05 was considered statistically significant Data expressed as median (IQR) and illustrated with box and whisker plots OBA: Opioid-Balanced Anesthesia; OFA: Opioid-Free Anesthesia

A total of 48 patients were recruited in the study and analyzed (Figure 1). Age, body mass index (BMI), ASA physical status, comorbidities, anesthesia duration, surgical duration, and hemodynamic parameters were comparable between the OFA and OBA groups (Table 1, Figure 2).

**Table 1:** Comparison of Patient characteristics and Procedure Duration between OBA and OFA Groups ASA: American Society of Anesthesiologists; DM: Diabetes Mellitus; HTN: Hypertension; n: Number of Patients; %: Percentage of Patients; IQR: Interquartile Range; BMI: Body Mass Index; OBA: Opioid-Balanced Anesthesia; OFA: Opioid-Free Anesthesia Data expressed as median (IQR) and n (%). A p-value less than 0.05 is considered significant * Mann-Whitney U test, # chi-square test

S no	Parameter	OBA (n=24)	OFA (n=24)	Test statistic	p- value
1	Age (years) median (IQR)	48 (42-58)	53 (41-59)	268*	0.68*
2	BMI median (IQR)	22 (19.25-24)	23 (21-25)	233*	0.258*
3	Anaesthesia Duration (minutes) median (IQR)	170 (148.5-185)	185.5 (162.25-214.25)	184*	0.148*
4	Surgery Duration (minutes) median (IQR)	143 (136-156)	139 (121.25-167.5)	286.5*	0.773*
5	ASA n (%) 1 2	17 (70.8) 7 (29.2)	16 (66.6) 8 (33.4)	0.097^#^	0.758^#^
6	Comorbidity n (%)			2.03#	0.808^#^
	No comorbidity	17 (70.8)	16 (66.6)		
	DM	3 (12.5)	1 (4.16)		
	HTN	2 (8.33)	4 (16.66)		
	DM+HTN	1 (4.16)	1 (4.16)		
	Others (asthma, respiratory)	1 (4.16)	2 (8.33)		

**Figure 3:**
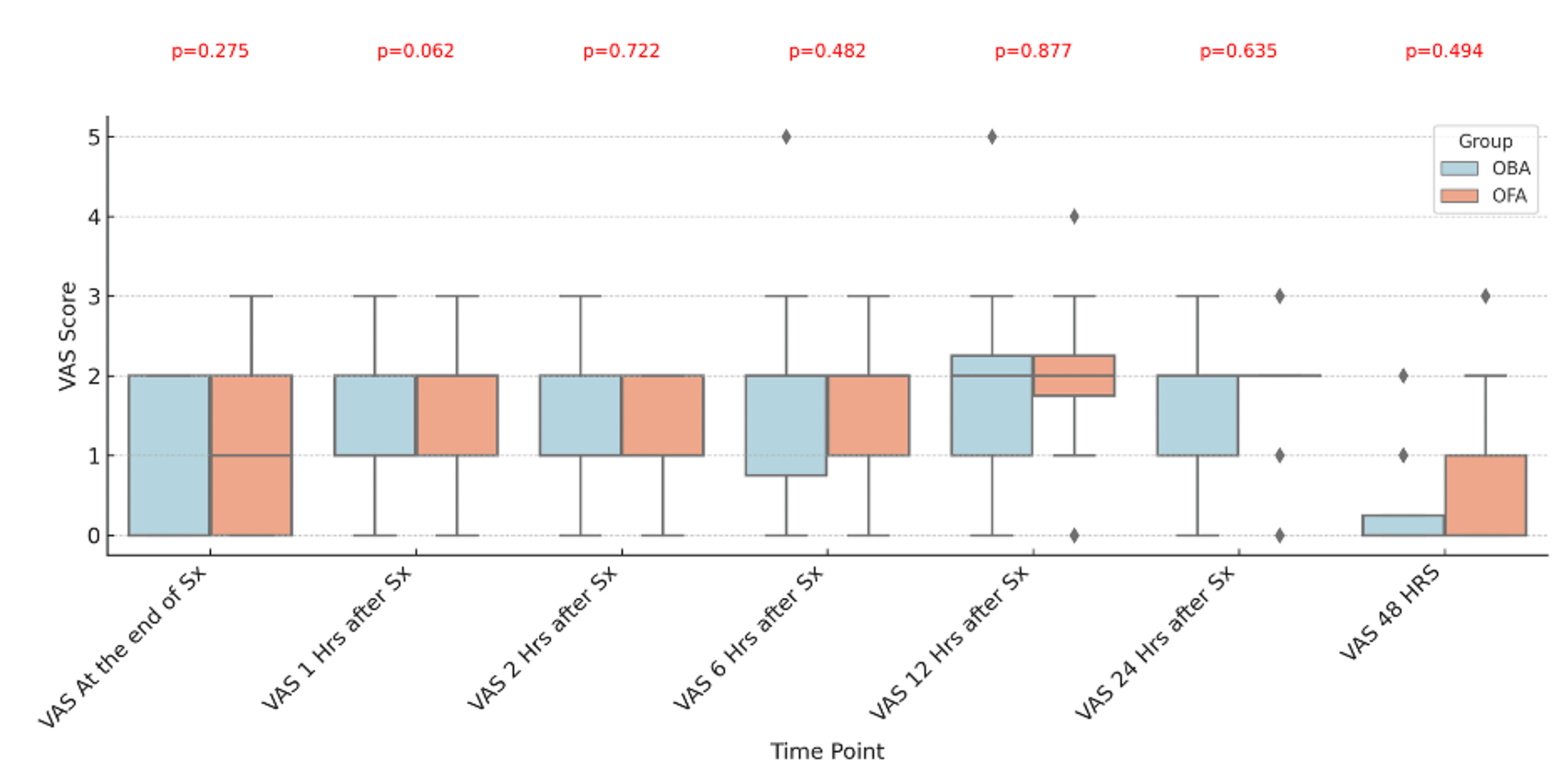
Comparative Analysis of Visual Analog Scale (VAS) Scores in OBA and OFA Groups from the Baseline to 48 Hours After Surgery A Mann-Whitney U test was applied, and a p-value less than 0.05 was considered statistically significant Data expressed as median (IQR) and illustrated with box and whisker plots OBA: Opioid-Balanced Anesthesia; OFA: Opioid-Free Anesthesia

Figure 3 shows the VAS score after the procedure, with an insignificant p-value at all time points.

Table 2 shows the quality of recovery for individual parameters at 24 hours and composite scores at 24 and 48 hours. The median (IQR) QoR-15 score was 130 (128-132.75) in the OBA group and 132.5 (132-135) in the OFA group (p = 0.054) at 24 hours and 142 (141-145) in the OBA group vs 145 (140.25-146) (p = 0.367) at 48 hours.

**Table 2:** Comparison of the Quality of Recovery (QoR-15) Score between OBA and OFA Groups IQR: Interquartile range; hrs: Hours; OBA: Opioid-Balanced Anesthesia; OFA: Opioid-Free Anesthesia Data expressed as median and interquartile range. *Mann-Whitney U test:  A p-value less than 0.05 was considered statistically significant.

S. no	Parameter	OBA (n=24)	OFA (n=24)	Mann-Whitney U test	p-Value *
QoR-15 item score at 24 hours					
1	Able to breathe easily	9 (8.25-9)	9 (8-9)	232	0.206
2	Been able to enjoy food	8 (8-8)	8 (8-8.75)	247	0.327
3	Feeling rested	8 (8-8)	8 (8-8.75)	273	0.691
4	Have had a good sleep	7 (7-8)	7 (7-8)	268	0.639
5	Able to look after personal toilet and hygiene	7.5 (7-8)	8 (7-9)	205	0.066
6	Able to communicate with family or friends	10 (9-10)	9.5 (9-10)	283	0.898
7	Getting support from hospital doctors and nurses	10 (9-10)	10 (9-10)	278	0.798
8	Able to return to work or usual home activities	9 (8- 9)	9 (9-9)	206	0.058
9	Feeling comfortable and in control	8 (8- 9)	9 (8- 9)	210	0.078
10	Having a feeling of general well-being	9 (8- 9)	9 (8- 10)	277	0.813
11	Moderate pain	9 (8- 9)	9 (8- 9)	280	0.853
12	Severe pain	10 (10- 10)	10 (10- 10)	276	0.555
13	Nausea or vomiting	10 (10- 10)	10 (9.25- 10)	280	0.822
14	Feeling worried or anxious	9 (8- 9)	9 (8- 9)	243	0.282
15	Feeling sad or depressed	9 (8- 10)	9 (8- 10)	280	0.852
QoR-15 total score					
1	QoR-15 at 24 hrs	130 (128-132.75)	132.5 (132-135)	196	0.054
2	QoR-15 at 48 hrs	142 (141-145)	145 (140.25-146)	245	0.367

**Figure 4:**
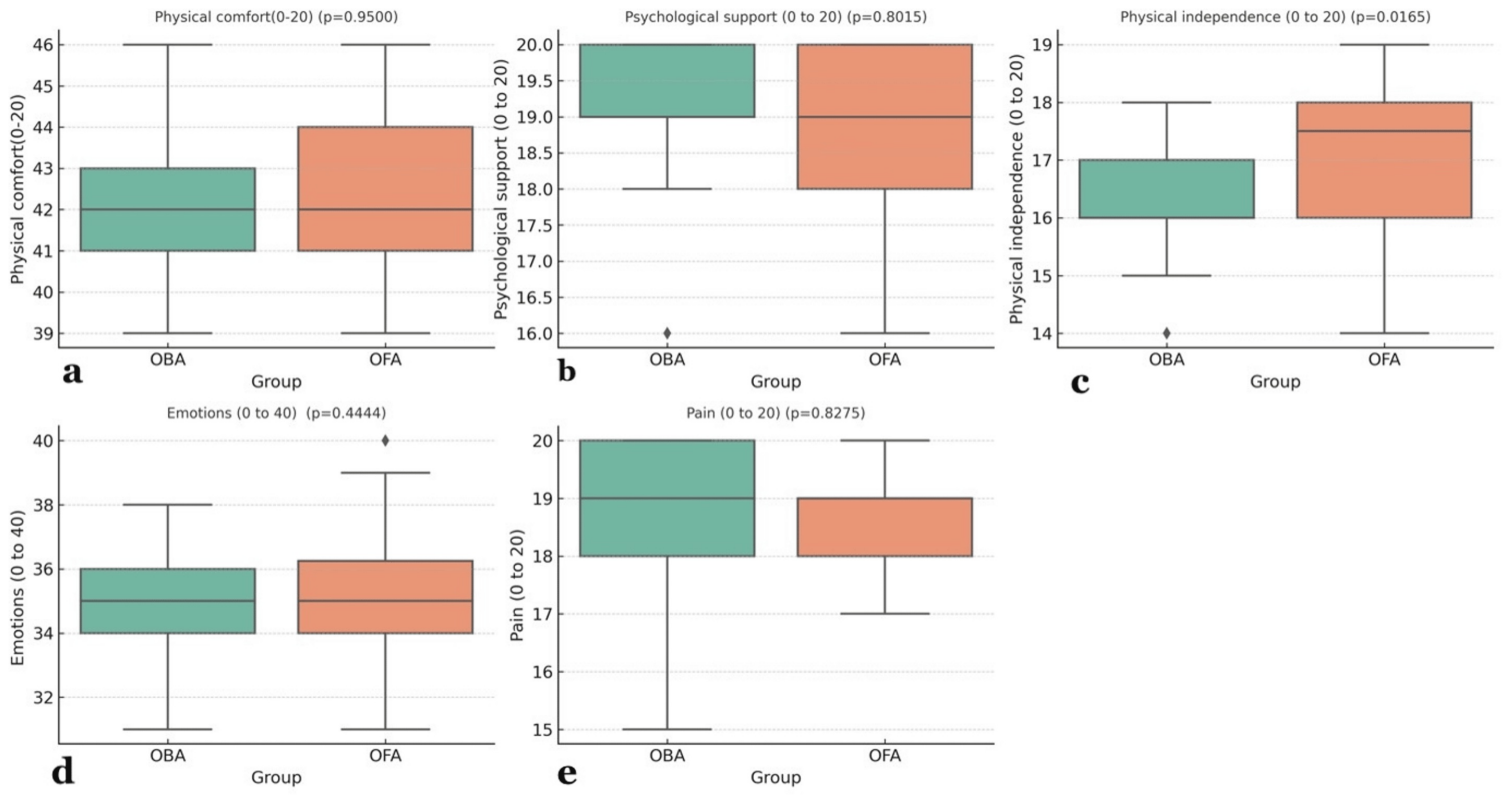
Intergroup Comparison of the Five Domains of Quality of Recovery (QoR-15) Scores Between OBA and OFA at 24 Hours a: Physical comfort, b: Psychological support, c: Physical independence, d: Emotions, e: Pain A Mann-Whitney U test was applied, and a p-value less than 0.05 was considered statistically significant Data expressed as median (IQR) and illustrated with box and whisker plots OBA: Opioid-Balanced Anesthesia; OFA: Opioid-Free Anesthesia

The QoR-15 in the OFA group had a higher median (IQR) for physical independence, 17.5 (16-18), against 16 (16-17) in the OBA group, with a p-value of 0.016. The p-values for physical comfort (P = 0.95), psychological support (p = 0.8), emotions (p = 0.444), and pain (p = 0.082) were statistically insignificant at 24 hours (Figure 4).

Two patients (8.4 %) in the OBA group and one patient (4.2%) in the OFA group required one dose of rescue analgesia (p = 0.683). Four patients (16.6%) in the OBA group had PONV and none in the OFA (p = 0.037). None of the patients in either group required secondary rescue analgesia or developed urinary retention, respiratory depression, or any other adverse events.

## Discussion

The QoR-15 score, a validated patient-centered outcome measure, is advocated as a standardized tool to evaluate patient comfort and recovery after surgery [8].

In this randomized study, a comparison of OFA and OBA showed no difference in the QoR-15 scores at 24 hours (p=0.054), 48 hours (p=0.367), and in the intraoperative hemodynamic parameters (HR, MAP). Out of the five domains of QoR-15, physical independence (p=0.0165) was found to be better with OFA, while the remaining four domains were comparable between the OFA and OBA groups. The VASs at all time points and requirements for rescue analgesia were comparable between the two groups. The incidence of PONV was significantly less in the OFA group.

The median (IQR) QoR-15 score 24 hours after surgery is 130 (128-132.75) in the OBA group and 132.5 (132-135) in the OFA group, which is consistent with a study by Zhang et al. However, our study differs due to the use of transdermal fentanyl patch in the OBA group and local anesthetic infusion for 72 hours into the paravertebral space in the OFA group, monitoring QoR-15 score for 48 hours [9].

The comparison of QoR-15 scores and hemodynamic parameters showed OFA is equivalent to OBA in terms of patient satisfaction and intraoperative hemodynamic parameters. Similar conclusions were drawn in a study by Kalagara et al., where OFA was found to be equally effective compared to OBA for postoperative pain assessed in terms of VAS [10].

Unlike the meta-analysis by Yu et al., which showed the OBA group to have a greater requirement for rescue analgesia compared to the OFA group, the total number of patients requiring rescue analgesia in our study was comparable between the two groups. This could be due to the use of fentanyl patches in the OBA group, which provided adequate postoperative analgesia [11].

A comparison of VAS scores shows no significant difference between the two groups; 95.8 % of the OFA group had VAS less than three at all the time points, which is consistent with the findings of a study by Raghupathy et al., in which OFA with regional techniques was compared with OBA in laparoscopic surgeries [12].

Four patients (16.6%) in the OBA group and none in the OFA group developed PONV. A similar reduction in the incidence of PONV with the administration of TIVA while avoiding opioids was found in a study conducted in bariatric surgeries [13]. A meta-analysis focused on OFA by Olausson et al. found an advantage of minimizing PONV while providing similar analgesic effects with OFA, similar to the findings of our study. They have concluded a need for the formulation of different non-opioid-based protocols [14]. OFA using local anesthetic infusion for regional nerve block in the postoperative period, with the advantage of avoiding opioid-related side effects such as PONV, while providing analgesia and quality of recovery comparable with OBA, can be a better choice for patients diagnosed with breast carcinoma coming for modified radical mastectomy surgeries.

The strength of our study is the provision of multimodal analgesia in both groups. Local anesthetic infusion into the paravertebral space for 72 hours in the postoperative period could have maintained comparable QoR-15 scores in the OFA group, though the non-opioid analgesic plan was adopted. Studies on OFA that utilized paravertebral block can be seen in the literature, but unlike the continuous infusion followed in our study, the previous studies have given a single dose of local anesthetic during the block [15]. The OBA group received transdermal fentanyl patches in addition to the regular intravenous analgesics for post-operative analgesia. A meta-analysis on OFA showed results consistent with our study for analgesia and PONV, however, none of the analyzed studies used a transdermal fentanyl patch or continuous infusion for the paravertebral block [2,9]. Apart from the VAS for pain, we have assessed the QoR-15 score, which has five domains to cover the physical, psychological, and emotional components in addition to pain. The follow-up for 48 hours in the postoperative period further adds strength to our findings.

The study has a limitation of not having a control group, which receives a placebo in paravertebral block followed by OBA which was not possible due to ethical concerns. Double blinding, where the patient and person performing an intervention need to be blinded, was not possible because placing a placebo patch or injecting a placebo after reaching the paravertebral space is unethical. However, observer bias was avoided by blinding the outcome assessor to group allocation and not involving the treating anesthetist in the outcome assessment.

In the future, similar studies comparing OFA with OBA in different major surgeries, such as laparotomies and thoracotomies, which usually require large doses of opioids for analgesia, can add to the applicability of the findings of this study on a broader scale. Studies comparing different non-opioid modalities for analgesia while giving OFA can improve the options for OFA.

## Conclusions

OFA provides a better quality of recovery in the physical independence domain of the QoR-15 score compared to OBA. OFA is similar to OBA, considering the overall quality of recovery according to the QoR-15 score, postoperative analgesia, and intraoperative hemodynamic stability, with a decreased incidence of PONV.
